# Dental and Oral Problem Patterns and Treatment Seeking Behavior of Geriatric Population

**DOI:** 10.2174/1874210601711010230

**Published:** 2017-04-28

**Authors:** Bader K. AlZarea

**Affiliations:** College of dentistry, Al-Jouf University, Al-Jouf, Saudi Arabia

**Keywords:** Geriatric, Oral health, Behavior, Saudi Arabia, Dental patterns, Dental care

## Abstract

**Background:**

The manifestations of oral changes and disorders affecting the geriatric population are different from the rest of the population. Inaccessibility to dental care is a compelling impediment to avail oral health services.

**Objective:**

The aims were to assess the dental and oral problems and to find out the determinants of oral health seeking behaviour among elderly population of Al-Jouf province, Saudi Arabia.

**Methods:**

The present cross sectional study included geriatric patients of 60 years and above, who visited the College of Dentistry, Al-Jouf University. A simple pre-structured questionnaire was filled by the patients, which comprised of demographic details and the different oral complaints of elderly and the type of health care utilized for those complaints.

**Results:**

Out of total 892 elderly persons included, 51.79% were males and 48.21 were females. The most common oral problem was missing tooth (78.69%) followed by gum problems (74.21%). 39.5% males and 28.0% females visited general dental practitioners for oral health care. Majority of the participants (32.8%) suggested accessibility as a basic factor in determining the health care source. The difference in the distribution of male and females or association between the type of care and gender and distribution for choosing a health care source was found to be statistically significant (*p* < 0.05).

**Conclusion:**

Inaccessibility to dental care emerged as an important barrier to avail oral health services. Adequate access to medical and dental care can reduce premature morbidity and mortality, preserve function, and enhance overall quality of life.

## BACKGROUND

Bureau of Health Profession defines “elderly” as “a population with health care conditions and needs, which differ significantly from those of younger people, which are often complicated by the physical, behavioral, and social changes associated with aging. This would include all persons over the age of 60 years, but may include slightly younger people who are subject to similar physical and/or mental conditions [1]. Due to improvements in health technology and increase in life expectancy, a significant number of older people are becoming consumer of health resources [2]. The manifestations of ill health in elderly people are distinct from the rest of the population. In a study conducted on health status and health seeking behavior of elderly persons (aged 65 years and above), in Dagoretti division, Nairobi Kenya, it was found that 40% suffered from dental problems [3]. In another recent study conducted by World health organization in collaboration with the Government of India to find the health problems of elderly population (aged 60 years and above), it was found that a large proportion *i.e.*, 32.6% (133/407) had dental problems apart from other medical problems like diabetes, hypertension and ischemic heart disease [4]. These findings are sufficient to raise the question ‘Why the elderly population suffers from a significant percentage of medical and dental problems? This segment of population has certain physical and financial constraints that impede timely utilization of available health care services, like increased distance from health care provider, cost of treatment, transportation, long waiting time, not having medical insurance, *etc* [5, 6].

The proportion of Canadian adults aged ≥ 65 years who visited a dentist in the preceding 12 months was reported to be 41.1% in the 2000-01 Canadian Community Health Survey [7]. Good oral health is known to be an integral part of general wellbeing and a contributory factor to the quality of life [8]. But a patient considers several factors before he/she chooses and/or visits a dentist. These factors may be patient related/internal factors or service provider related/external factors. Another classification of such factors is that they may act as barriers or enablers in the utilization of dental services. A comprehensive list of such factors is the cost of treatment, location/accessibility of dental clinic, waiting hours, patients beliefs on dental health, competence/professionalism of dentist, ethnicity, education status of patient, previous dental experience, fear of dental treatment, functional and medical status of individual (those living in long term facilities), nature of dental complaint, lack of dental insurance, friendly clinic environment, *etc* [7-10]. Some authors have also divided these factors into predisposing factors, enabling factors and need based factors. Literature supports a number of articles incorporating these different factors to find out why elderly population does not use dental services often [1, 7,11-13]. Authors have reported that the main barrier for oral health care is the perceived need. Majority of elderly avoid dental visits because they do not see a need for treatment [14]. The objective of the present study was to assess the dental and oral problems and to find out the determinants of oral health seeking behaviour among elderly population (60 years and above) of Aljouf province, Saudi Arabia.

## METHODS

In the present cross-sectional study, data were collected from patients attending the Out Patient Department (OPDs) of College of Dentistry, Al-Jouf University. Prior clearance was obtained from the institutional ethical committee. The study sample included geriatric patients who consented and belonged to the working age group of 60 years and above and who visited the OPD, for routine check-up, scaling, with complain of pain, for prosthesis. Non probability purposive sampling technique was used to induct patients in the study. A simple pre-structured questionnaire was filled by the patients in OPD which comprised 2 basic components. First component asked about their demographic data (Name, age, gender, education status, economic status and source of history and reliability of historian *etc.*) and the second component addressed the different oral complaints (Pain, mobility of teeth, halitosis, edentulism, difficulties in speech and mastication, soft tissue lesions, *etc.*) of elderly and the type of health care utilized (Scaling, restoration, extraction, prosthesis, *etc.*) for those complaints. They were also inquired about the factors that they considered important while choosing dental service (Nature of complaint, accessibility, cost, quality of service, previous experience). The responses to all the inquiries ranged from very high to very low. The questions asked were related to what patients would prefer while selecting a dental facility. Data was analyzed using SPSS and statistical analysis was done using the Chi-square test and was considered to be significant at 5% level of significance.

## RESULTS

The distribution of elderly respondents according to age and gender is presented in Table **1**. Out of the total 892 elderly persons, 51.79% were males and 48.21% were females. Furthermore, out of 460 males, a maximum of 134 (29%) male respondents belonged to the age group of 60-64 years and a minimum of 54 (11.7%) males belonged to >80 years of age group followed by other age groups. Similarly, out of 430 females, a maximum of 118 (27.4%) female respondents belonged to the age group of 60-64 years and a minimum of 38 (8.9%) belonged to >80 years of age group followed by other age groups.

Table **2** represents the distribution of elderly respondents according to different complaints. 81.2% of males reported missing tooth complaint as compared to 74.5% females. The difference was found to be statistically significant (Chi-square = 5.5602, *p* = 0.0180). A significant and higher percentage of males (68.3%) had gum problems when compared to females (63.9%) at 5% level of significance (Chi-square = 4.2265, *p* = 0.0400).

A significant and higher percentage of females (62.5%) had xerostomia in comparison to males (51.1%) at 5% level of significance (Chi-square =12.5382, *p* = 0.0001). 21.6% of males reported to have facial pain in comparison to 36.3% in females. The difference was found to be statistically significant (Chi-square = 24.7602, *p* = 0.0001).

Table **3** reveals that, out of 460 males, a maximum of 184 (39.5%) male respondents visited general dental practitioners and a minimum of 22 (4.8%) males were using home remedies/other system of medicine followed by other sources of care. Similarly, out of 430 females, a maximum of 122 (28.0%) female respondents were visiting general dental practitioner and a minimum of 28 (6.5%) were visiting medical practitioners followed by other sources of care. The difference in the distribution of male and females or association between the type of care and gender was found to be statistically significant (Chi-square = 57.9062, *P* = 0.0001) at 5% level of significance.

Determinants of health care seeking behavior among elderly respondents have been shown in Table **4**. Out of 460 males, a maximum of 138 (29.6%) male respondents reported that accessibility was a prime factor in choosing the health care source and a minimum of 36 (7.7%) males revealed their previous experiences as determinant of health care seeking followed by other types of determinants. Similarly, out of 430 females, a maximum of 138 (29.6%) female respondents informed accessibility as a basic factor in determining the health care source and a minimum of 16 (3.7%) of females reported their previous experience as determinant of health care seeking behavior followed by other types of determinants. The difference of distribution of male and females is found to be statistically significant (Chi-square = 35.9473, *P* = 0.0001) at 5% level of significance.

## DISCUSSION

Poor oral health can adversely affect the quality of life by lowering the level of psychosocial well being and life satisfaction particularly among elderly people [13, 14]. Older persons are at risk of chronic diseases of the mouth, like dental caries, periodontitis, tooth loss/edentulism, benign mucosal lesions, oral cancer, xerostomia, oral candidiasis (acute pseudomembranous candidiasis, denture stomatitis and angular cheilitis). Oral and general health are also linked, suggesting a relationship between periodontal disease and diabetes, cardiovascular disease, pneumonia, rheumatologic diseases, and wound healing [15, 16]. Many older persons are prone to tooth loss because of periodontal detachment, caries (especially root caries) and gingival recession exposing cementum. Tooth loss is directly associated with mastication and nutritional problems. A peak in the incidence of severe tooth loss has been reported at around 65 years of age [17]. In the present study we recorded one or more missing teeth and not complete tooth loss/edentulism as increasing number of older persons are retaining some or all their teeth because of improvements in oral health care. In the present study we found that more number of males had missing tooth and gum problems (81.2% and 68.3% respectively) as compared to females (74.5% and 63.9% respectively). The results are similar to other studies which have reported that periodontal problems and tooth loss was high in males [18-20]. These authors have reported that greater periodontal destruction in males is because of more calculus, periodontal pocket and association with smoking, and later leads to loss of teeth. Reduced sensitivity of teeth with old age is due to receded pulp horns, pulp fibrosis, deposition of secondary or tertiary dentin, and decreased permeability of dentin. We recorded both root caries and coronal caries in tooth decay and found that gender distribution was not statistically significant. Mac Donald (2006) has reported that incidence of root caries in patients older than 60 years is twice that of 30 year olds [15]. In a study conducted in China, the prevalence of root caries was found to be significantly higher (43.9%) in elderly as compared to middle aged population (13.1%) [21]. In another reported study on very old persons 64% of persons older than 80 years had root caries, and up to 96% had coronal caries [22]. Risk factors for caries in older people are decreased salivary flow rate, institutionalization, lack of routine dental care, low socioeconomic status and poor oral hygiene. Two oral problems which were found to be present more in females were xerostomia and facial pain. The different factors causing xerostomia in elderly are, hypofunction of salivary glands, medications (especially antihypertensives, antidepressants, and antipsychotics) and manifestation of systemic diseases like diabetes. Polypharmacy is also common in elderly people and if the medications are xerostomia inducing drugs then this may result in a significant reduction in salivary flow rate [23]. In a study it was found that age, medication and female gender were significant risk factors for xerostomia [24]. Elderly people with xerostomia and tooth loss may have reduced masticatory ability leading to malnutrition. Malnutrition acts as a risk factor for periodontal disease due to lowering of body’s immunity. In turn, periodontal disease increases the risk of root caries and further tooth loss completing this vicious cycle.

Regarding consulting a doctor for oral complaints it was found that most elderly (39.5% males and 28% females) consulted a general dental practitioner, followed by dental specialist (23.2% males and 19.8% females). In a study to determine the rate of use of dental services by independently living older dentate and edentulous adults, out of 1751 participants 383 (21.9%) reported having visited a dentist in the past 6 months. The visitation rate for dentate seniors was significantly higher than that for edentulous [7]. In another study 94% of the edentulous reported visiting only for pain or trouble compared to 26% of the dentate [13]. Such low visitation of dentist by edentulous people actually decreases the overall rate. U.S. Preventive Services Task Force recommends regular dental visits for persons aged greater than or equal to 65 years [1]. It was found that visitation rates were higher for males compared to females and more number of females preferred home remedies comparatively in the present study. This may be due to males suffering from more oral complaints as compared to females hence likely to visit dentist often. Also females are found to be significantly more dentally anxious than their male counterparts which results in longer intervals between dental visits [25]. Both males and females cited accessibility as major determinant factor followed by quality of services for seeking oral health care. Now inaccessibility covered both lack of transport services and non availability of a dentist. This is similar to other studies which also cited accessibility of a dental clinic as one of major factor for seeking/not seeking dental care [14, 26]. The functional and medical status of elderly makes them unable to go long distances to seek medical or dental care. But other studies found different factors which act as barriers like high cost of treatment, fear of dental care not having dental insurance and dental [12, 14, 15, 26, 27]. Lack of perceived need for oral care has often been cited as an important barrier to older adults receiving dental services [27, 28]. Many older adults accept chronic disease as an inevitable and even normal part of the aging process and hence see no need for treatment. A study has reported that poor self perceived oral health and relatively poor quality of life co-exist in the same subgroup of older adults [29]. Previous dental experience was the last determinant for seeking oral health as older adults report less painful experiences for dental procedure.

## CONCLUSION

The present research aimed to investigate the response of elderly people to dental and oral diseases and the reasons for that response. Inaccessibility to dental care emerged as an important barrier to avail oral health services. Majority of elderly subjects reported visiting private practitioners during illness due to easy accessibility. They also preferred good quality of service and did not consider economic status as a hindrance in the utilization of dental care.

## Figures and Tables

**Table 1:** Distribution of elderly respondents according to age and sex.

**Complaints**	**Male**	**Female**	**Chi-square**	***P*-value**
Missing tooth	378 (81.2)	324 (74.5)	5.5602	0.0180*
Gum problems	352 (75.6)	310 (71.3)	4.2265	0.0400*
Tooth decay/Pain	318 (68.3)	278 (63.9)	1.7553	0.1852
Xerostomia	238 (51.1)	272 (62.5)	12.5382	0.0001*
Sensitivity in teeth	228 (49.0)	190 (43.7)	2.3861	0.1223
Fracture/broken tooth	202 (43.4)	162 (37.26)	3.3731	0.0663
Facial pain	99 (21.6)	157 (36.3)	24.7602	0.0001*
Soft tissue lesions in mouth	92 (19.7)	74 (17.0)	1.0753	0.3001

*P < 0.05

**Table 2:** Distribution of elderly respondents according to the complaints.

**Type of care utilized**	**Male**	**Female**	**Total**
General dental practitioner	184 (39.5)	122 (28.0)	306 (33.7)
Dental specialist	108 (23.2)	86 (19.8)	194 (21.5)
Dental college	72 (15.5)	52 (12.0)	124 (13.8)
Self medication/OTC	36 (7.7)	66 (15.4)	102 (11.6)
Medical practitioner	40 (8.6)	28 (6.5)	68 (7.6)
Home remedies/Other system of medicine	22 (4.8)	76 (17.6)	98 (11.2)
Total	462 (100)	430 (100)	892 (100)

Chi-square = 57.9062, *P* = 0.0001*

**Table 3:** Types of health care utilized for reported complaints among elderly respondents.

**Determinants**	**Male**	**Female**	**Total**
Accessibility	138 (29.6)	156 (36.0)	294 (32.8)
Quality of service	122 (26.3)	104 (24.0)	226 (25.2)
Nature of complaint	76 (16.3)	46 (10.6)	122 (13.5)
Education status	42 (9.0)	82 (18.9)	124 (14.0)
Economic status	48 (10.3)	26 (6.0)	74 (8.1)
Previous experience	36 (7.7)	16 (3.7)	52 (5.7)
Total	462 (100)	430 (100)	892 (100)

Chi-square = 35.9473, *P* = 0.0001*

**Table 4:** Determinants of oral health care seeking behavior among elderly respondents.

**Age groups**	**Male n (%)**	**Female n (%)**	**Total n (%)**
60-64yrs	134 (29)	118 (27.4)	252 (28.3)
65-69 yrs	114 (24.7)	116 (27)	230 (25.8)
70-74 yrs	104 (22.5)	112 (26)	216 (24.2)
75-79 yrs	56 (12.1)	46 (10.7)	102 (11.4)
>80 yrs	54 (11.7)	38 (8.9)	92 (10.3)
Total	462 (100)	430 (100)	892 (100)

Chi-square = 4.4729 *P* = 0.3451
